# The epidemiology of attacks on statues: New Zealand as a case study

**DOI:** 10.1371/journal.pone.0252567

**Published:** 2021-06-03

**Authors:** Nick Wilson, Amanda C. Jones, Andrea Teng, George Thomson

**Affiliations:** Department of Public Health, University of Otago Wellington, Wellington, New Zealand; Neijiang Normal University, CHINA

## Abstract

**Objectives:**

We aimed to describe the epidemiology of statue attacks along with statue representativeness relative to modern day demographics in one case study country: New Zealand.

**Methods:**

We performed Internet searches for the existence of outdoor statues of named individuals and historical attacks in New Zealand (NZ), combined a national survey with field visits to all identified statues to examine for injuries and repairs.

**Results:**

Of the 123 statues identified, nearly a quarter (n = 28, 23%) had been attacked at least once (total of 45 separate attack events), with the number of attacks increasing from the 1990s. Attacks involved paint/graffiti (14% of all statues at least once), nose removal/damage (7%), decapitation (5%), and total destruction (2%). The risk of attack was relatively higher for statues of royalty (50%), military personnel (33%), explorers (29%), and politicians (25%), compared to other reasons for fame (eg, 0% for sports players). Statue subjects involved in colonialism or direct harm to Māori (Indigenous population), had 6.61 (95%CI: 2.30 to 19.9) greater odds (adjusted odds ratio) of being attacked than other subjects. Most of the statue subjects were of men (87%) and Europeans (93%). Other ethnicities were 6% Māori (comprising 15% of the population) and 1% each for Asian and Pacific peoples, who comprise 12% and 7% of the population respectively.

**Conclusions:**

This national survey found an association between statue attacks and the role of statue subjects in colonialism or direct harm to the Indigenous population. Furthermore, the demography of the statue subjects may represent historical and current social power relationships—with under-representation of women and non-European ethnic groups.

## Introduction

Around the world statues are being attacked, critiqued or removed from prominent public settings. In particular, statues commemorating Confederate leaders and soldiers of the American Civil War are now regularly attacked or removed [1]. Statues of Indigenous peoples that are portrayed in a racist way are also being retired [2], along with those considered sexist (eg, the “Civic Virtue” statue in New York City), and a statue of a famous doctor who committed experiments on slaves (J Marion Sims) [3]. Historical political grievances have resulted in a Ukrainian Parliament law requiring the removal of statues to Lenin, Marx and Engels [4]. This has resulted in the removal of an estimated 5500 statues of Lenin, though two remain in the Chernobyl Exclusion Zone. Attacks on statues can occur over a long period, with one of William III (of Orange) being damaged or numerous occasions after 1710 and finally destroyed in 1928 [5].

Disputes over statues have soured both domestic and international relations. For example, statues of “comfort women” have strained relationships between Japan and South Korea [6], and also between Japan and the USA [7]. A statue of Alexander the Great has contributed to tension between North Macedonia and Greece—albeit with recent signs of resolution [8]. More dramatically, the removal of a Red Army Soldier statue in Estonia appears to have triggered two nights of riots and a major cyberattack on the country [9]. A giant statue in India that cost $US 430 million has triggered debate over the misuse of resources [10], and has been criticised for being threatening to non-Hindu citizens [11]. One direction in the debates on contentious memorials is seen in a Northern Ireland local policy that “all council buildings and facilities should be neutral environments in terms of how they reflect political or religious opinion” [12].

Research on the disputation around statues can be considered a subset of the study of iconoclasm, where the many motives for attacks on images are considered. The process of attack or dispute may be a reflection of broader conflict in societies, and thus who has the power on how images are used [13], or how the “symbolic landscape” is created [12].

Given this background, we first aimed to explore the epidemiology of statue attacks in one case study country: New Zealand. This is a country where, the conflicts around statues have included concerns about nudity [14], colonisation [12], and the commercial associations of public sculptures [15]. New Zealand is a country with around 700 years of settlement by Māori (the Indigenous people) and around 180 years of European settlement. It has had a history of colonial oppression and wars against Māori by Europeans, along with persisting injustices borne by Māori [16].

Our second aim was to describe the sex/ethnic distribution of the statue subjects to determine their representativeness relative to population demographics. Other population groups in New Zealand beside Māori, who have traditionally been relatively disempowered include: Pacific peoples, Asian peoples and woman in general (eg, the latter with persisting under-representation in Parliament and unequal pay) [17]. New Zealand currently has a diverse ethnic composition: Māori (14.9% of the population), Asian peoples (11.8%), and Pacific peoples (7.4%) [18].

## Methods

For the purposes of this study we included statues, busts and full-body bas reliefs of named people that had been erected in outdoor public settings in New Zealand and which were approximately life-sized or larger. We aimed to focus on statues of people in the modern-era (from the time of the explorer Abel Tasman’s first visit in New Zealand in 1642). Therefore, we excluded statues of subjects who were: unnamed generic figures (eg, mainly soldiers on war memorials); foundational religious figures (eg, Jesus, Madonna and various saints); pre-European ancestors of Māori; and fictional characters from literature and mythological figures. These exclusions were on the grounds of named figures potentially having a stronger historical context than generic figures, that a limitation to figures from after 1642 enabled a greater focus on current conflicts within society, and because data collection on each of these statues throughout the country was more feasible than for generic statues (given this was an unfunded study which involved extensive travel). There are over 500 World War One memorials alone, many with statues of generic military personnel [19]. Furthermore, Māori carvings of ancestors are typically highly symbolic and require interpretation by those with iwi (tribal) expertise, and there are over 110 iwi in New Zealand.

We restricted the sample to public settings, as this is where the prominence of the person represented by the statue is most clearly endorsed, in contrast to statues in places such as cemeteries, where it may particularly represent a family’s respect for the person. Additional details on our search strategy for identifying statues, field data collection, data coding and analysis are reported in the S1 File, along with a list of types of excluded statues in S1 Table in S1 File.

### Search strategy

Briefly, we compiled a list of existing statues by reviewing books and websites on New Zealand sculptures, memorials, monuments and statues. To supplement this list, we also performed searches in Google Images, Google Scholar and a national repository of all New Zealand newspaper issues (“Papers Past”) by combining variations of statue terminology (eg, statue, bust), geographic locations (eg, Zealand, Auckland) or other identifiers (eg, ‘Māori’, names of famous New Zealanders [20]). Subsequent to the original searches, we incidentally located six additional statues as a result of both further internet searches (n = 3), and field work (n = 3) up to the last inclusion date (13 April 2019 when the statue of Alex Lithgow was unveiled). This suggested an upper estimate of the sensitivity of our initial strategy at 94%.

Identifying a statue as per the above search strategies frequently revealed historical information about intentional and unintentional injuries to the statue. We performed additional searches in Google Images, Google Scholar and *Papers Past* with attack-related search terms (eg, statue and vandalism, statue decapitation). For all statues with any history of damage, we performed additional searches for information about the possible motives for such damage.

### Field data

Site visits to all the statues that were still outdoors were conducted between September 2018 and August 2019. For each statue, photographs were taken of the setting, all sides of the statue, and any associated plaques or information boards. In particular, the statues were closely examined for evidence of past and current injuries (including attacks with paint in which residual paint may be identified in hard to clean areas such as the ears and nose). Photographs allowed for any joint decision-making by the investigators on what was likely to be evidence of a past attack (but this was rarely required). Since all the statues were in public settings, no permits were required for the described study, which complied with all relevant regulations.

### Data coding and analysis

Data from internet searches and field visits were collated in an Excel file (available as S2 File) and analysed. The denominator used was for all statues unveiled at any time up to the last inclusion date (13 April 2019). This included all statues that were destroyed in attacks (n = 2) or from decay (n = 1), stolen (n = 1), or moved from outdoors and into interior settings (n = 14). If a stolen statue was replaced (n = 1), the replacement statue was counted as a new statue in the denominator. Each statue was coded for the following characteristics: time (eg, unveiling year), setting (eg, city), the statue subject’s general characteristics (eg, sex), the subject’s primary reason for fame (eg, politician), and the features of the statue (eg, statue material).

We defined a statue attack as being where there was documentation of an attack or from our field observations of obvious repairs, missing body parts (eg, noses), paint remnants, or graffiti. Attack documentation consisted of reports in *Papers Past* (a collection of New Zealand and Pacific text items) or in online media reports.

Our *a priori* hypothesis was that controversial statues could be at an increased risk of attack and that in the New Zealand context this would be statues of subjects involved in colonialism or direct harm to the Indigenous population. This was based on the grounds that such injustice may contribute to grievances that could contribute to statue attacks over long periods, based on international patterns of statue attack (see *Introduction*). The list of those statue subjects that fitted this category is detailed in the (S3 Table in S1 File). An *a priori* decision was also made to adjust the analysis for two obvious potential confounders: the age of the statue and its construction material (eg, marble being much easier to damage than bronze in terms of decapitation and nose loss etc).

Results were reported as simple frequency distributions but also logistic regression and Poisson regression analyses were conducted. The regression analyses adjusted for statue age (linear covariate) and statue material. We described results as significant below an alpha value of 0.05 (ie, p-value <0.05). Statistical analyses were conducted using EpiInfo, OpenEpi, and R (version 4.0, R Institute, Vienna, Austria).

## Results

### Description of the statues

**Fig 1:**
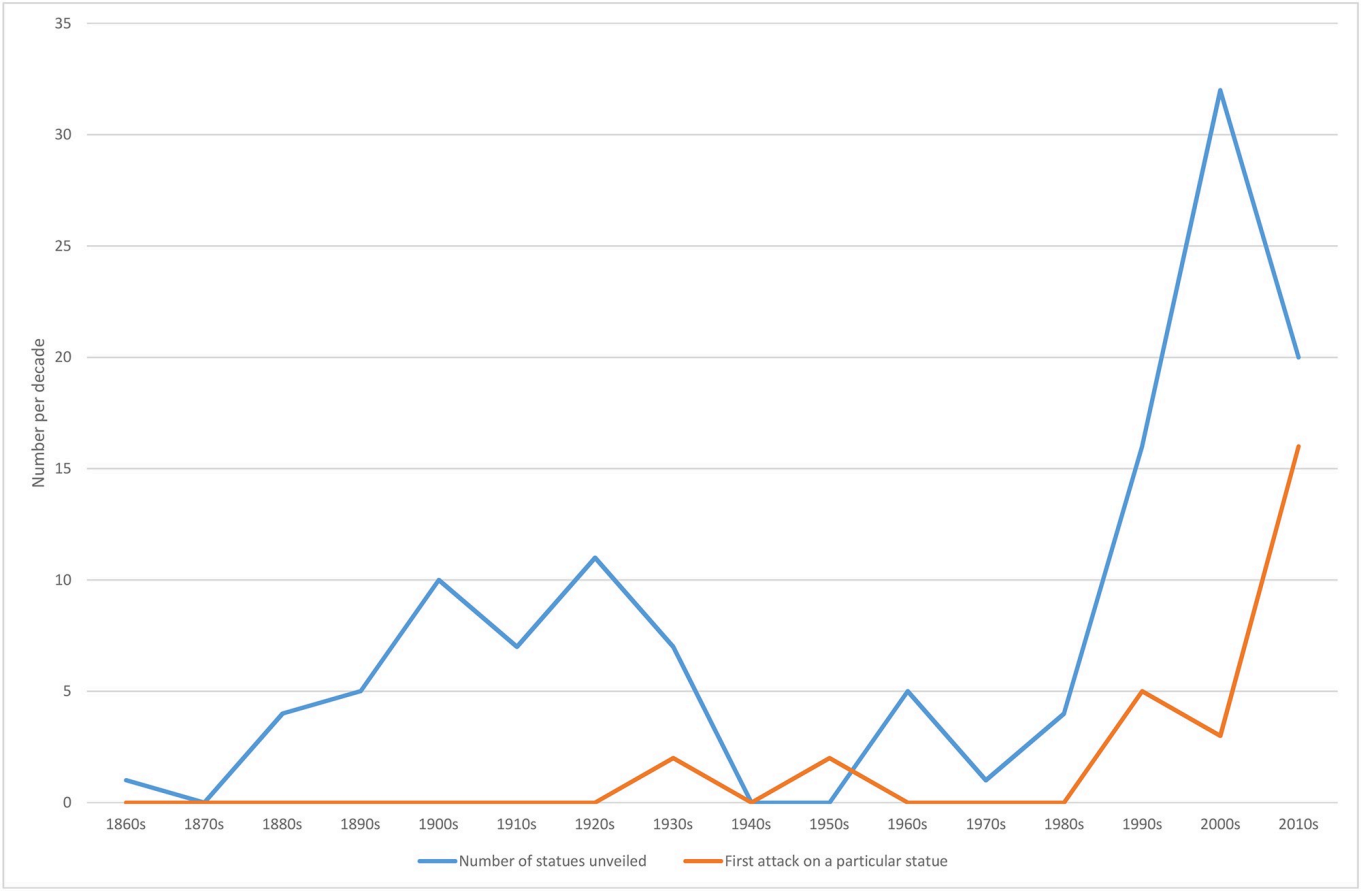
Number of statues of named individuals unveiled in public outdoor settings in New Zealand and first intentional attacks on each statue by decade. (Note: for the last decade, data collection was not quite complete, ie, up to the 13 April 2019 unveiling of the Lithgow statue).

We identified 123 outdoor statues of modern-era named people in outdoor public settings throughout New Zealand (Table 1; additional details in the S2 Table in S1 File). Since the first such statue was unveiled in 1867, there was a U-shaped pattern of statue unveiling, with an initial peak in the 1920s, no unveilings in the 1940s and 1950s, and then with increased unveilings from the 1990s onwards (Fig 1). These statues were primarily of men (87%) and Europeans (93%). Other ethnicities were 6% Māori and 1% each for Asian and Pacific peoples. The most common primary reason for notability in these statue subjects was being a military personnel (17%), then a politician (16%), and then an explorer or artist/writer (each 11%). There were more sports players (9%) than doctors (2%) or scientists (2%). Particular individuals also dominated: 24% of the statues were of people who had multiple statues ie, Captain Cook led with five; and Queen Victoria and Robbie Burns had four each.

**Table 1:** Characteristics of all the 123 statues of named people in outdoor public settings throughout identified in this national survey (New Zealand).

Characteristic	N	%	Comment
**Time**			
Unveiling year: median calendar year (interquartile range [IQR])	1993 (1923 to 2009)	–	See Fig 1 for the time trends by decade. Full range was from 1867 to 2019.
• Mode	2009	–	There were 15 statues unveiled in this year.
Statue age: mean (years)	47.0 (SD = 45.6)	–	From unveiling date to the end of the year 2018 if still standing (otherwise to date of destruction, theft or to being moved indoors).
• median (IQR)	25.5 (7.5 to 96.3)	–	As above.
Statue-years (cumulative)	5780	–	As above.
**Setting**			
• City	91	74.0%	
• Town	31	25.2%	
• Rural	1	0.8%	
Statue moved from outdoors to an indoor location	14	11.4%	Most of these (n = 12) were moved on a temporary basis to facilitate earthquake repairs on an adjacent building in Christchurch.
**Statue subject characteristics**			
Statues per subject			
• One	93	75.6%	
• Two or more	30	24.4%	The most frequent was for Captain Cook (n = 5).
Sex			
• Male	107	87.0%	
• Female	16	13.0%	
Ethnicity			
• European	114	92.7%	Of these, 13% (n = 15) were female.
• Māori	7	5.7%	Of these, 14% (n = 1) were female.
• Pacific peoples	1	0.8%	This single statue was of Sir Michael Jones, a rugby player (S3 Fig in S1 File).
• Asian peoples	1	0.8%	This was of Mahatma Gandhi. There were nil statues of Asian people who had lived in NZ.
On a list of 100 most famous New Zealanders [20]	19	15.4%	Includes those with multiple statues erected (eg, George Grey, Edmund Hillary, Peter Snell).
**Statue subject—primary reason for fame**			
• Military personnel*	21	17.1%	Included 4 Victoria Cross winners.
• Politician	20	16.3%	Prime ministers, members of parliament, mayors.
• Explorer	14	11.4%	Eg, Abel Tasman Fig 2.
• Artist/writer	13	10.6%	
• Sports player	11	8.9%	Eg, Sir Michael Jones, a rugby player (S3 Fig in S1 File).
• Business leader	10	8.1%	
• Founder (eg, of a town)	8	6.5%	
• Social activist	7	5.7%	
• Royalty	6	4.9%	King George V (n = 2) and Queen Victoria (n = 4).
• Other	13	10.4%	Religious leader (n = 2), doctor (2), Māori leader (2), scientist (2), local character (n = 2; “swagger” and legendary sheep rustler), and none of the above (ie, n = 3; architect, aviator, engineer/philanthropist).
**Statue features**			
Statue material			
• Bronze	84	68.3%	
• Other materials	39	31.7%	Included marble, granite, concrete.
No plaque (ie, unnamed statue)	3	2.9%	Rudyard Kipling, John Ruskin, Camille Malfroy. The denominator (n = 105) was the number of field observations.
Any Māori language text on the plaque or statue structure	7	5.7%	For any text in te reo Māori, but excluding the Māori name of the statue subject in one case.

* This was for the “primary” reason for fame.

Most statues were made of bronze (68%). Three statues had no associated name plaque and use of Māori language (te reo Māori) on the plaque or monument was uncommon (6%).

### Statue attacks

**Fig 2:**
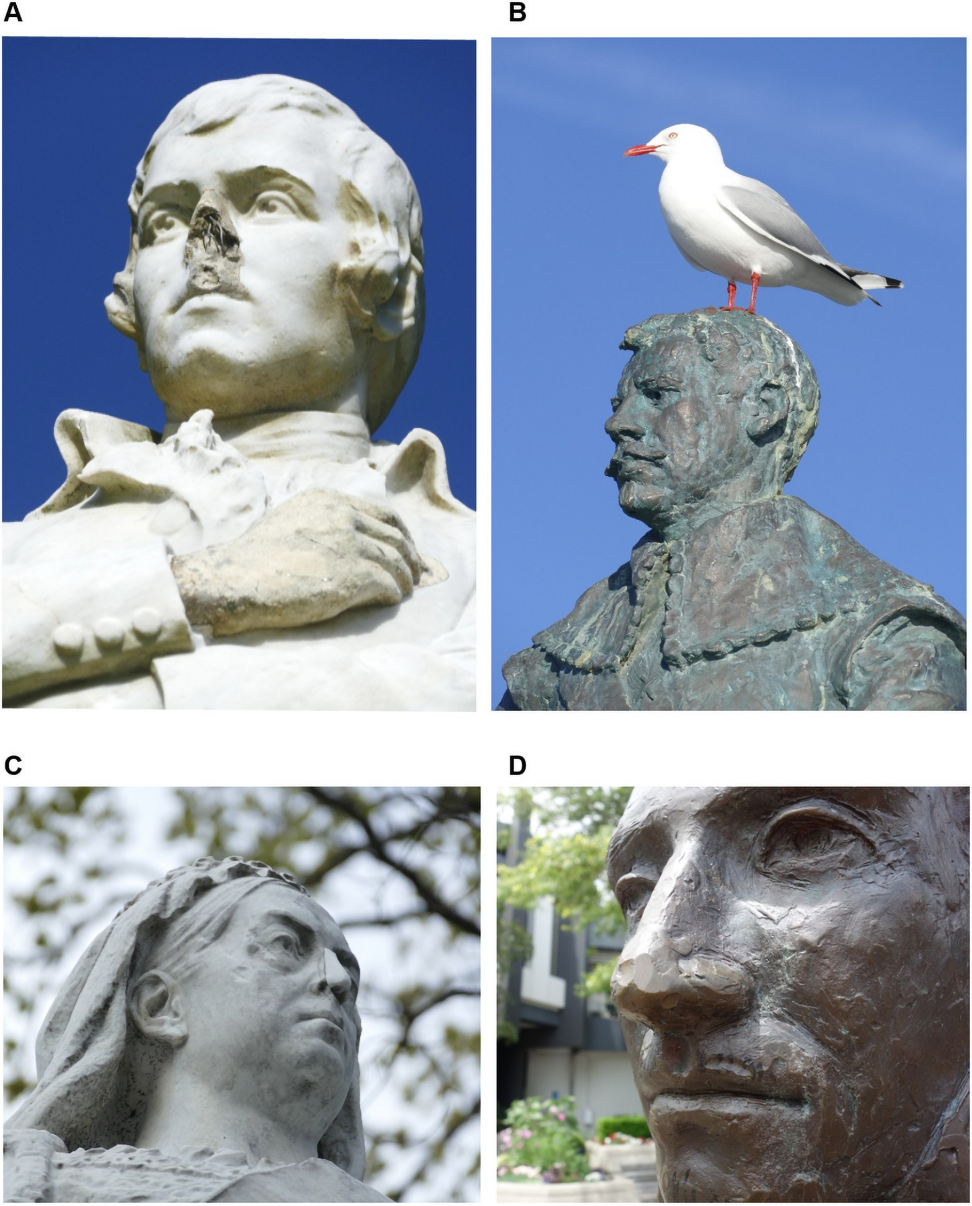
Examples of statues. Panel A: Statue of the poet Robbie Burns (Hokitika) showing nose damage and suboptimal hand repair. Panel B: Statue of an explorer Abel Tasman whose expedition killed Māori when arriving in New Zealand. Panel C: Statue of Queen Victoria (Dunedin) with a suboptimal nose repair. Panel D: Dented nose from an attack with a hammer on a statue of Captain Hamilton (Hamilton City); (all photographs by the 2^nd^ author, 2018/2019).

Of the 123 statues in the sample, 28 (23%) had been intentionally attacked at least once. Recorded statue attacks first began in the 1930s, but the number of attacks has risen since the 1990s (Fig 1). There were an estimated 45 separate attacks on 28 statues (mean = 0.37 attacks per statue out of all 123 statues, or 7.8 attacks per 1000 statue-years of existence, Table 2). The most common type of attack was a paint attack or graffiti (14% of all statues for any attack), followed by nose removal or damage (7%) (Fig 2 and S1 Fig in S1 File). Decapitation occurred a total of 11 times among six statues, with two statues decapitated multiple times (eg, five times for the statue of King George V at Matakana). Two statues were destroyed in attacks and one was stolen.

**Table 2:** Type of injuries from attacks on the statues of named figures in outdoor public settings in this national survey.

Type of intentional injury	N	%	Additional details
Any intentional physical damage or paint attack	28	22.8%	Some statues had multiple attacks ie, an estimated 42 separate attack events on these 28 statues (mean = 0.34 attacks per statue out of all 123 statues, or 7.3 attacks per 1000 statue-years of existence). See Fig 1 for the time trends of first attacks by decade.
• Any paint attack/graffiti	17	13.8%	Some of these attacks were not historically documented but were inferred from paint remnants on the statue from field observations. We included attacks with words painted on the statue and one attack with nugget (shoe polish). For statues that reported “multiple” paint attacks, we used 2 attacks per statue in the analysis. There were a total of 20 known paint attacks used in the analysis.
• Any nose removal/damage	8	6.5%	In most cases the nose was removed (eg, Fig 2, S1 Fig in S1 File). For 3 attacks on bronzes there was just denting of the nose. For one statue with reported “multiple” nose losses we used “2 attacks” in the analysis. There were a total of 9 attacks used in the analysis.
• Any decapitation	6	4.9%	Included statues: John Ballance (Whanganui) [twice]; John Ballance (Wellington); Robbie Burns (Timaru); King George V (Matakana) [five times]; George Grey (Auckland); Field Marshal Kitchener (Auckland). Two statues had multiple decapitations so there were 11 decapitations among these six statues.
• Complete destruction or removal	3	2.4%	Included statues: John Ballance (Whanganui), following a period of being headless; the bust of Admiral Tait (Timaru) was stolen; after decapitation, the remains of the Kitchener statue (Auckland) were taken down and the statue was never replaced. (Not included here is a statue of George Grey made of wood, which rotted away within several years of unveiling).
• Any other injuries	8	6.5%	These included the removal of a foot and hands, damage to fingers, denting of parts of the head (other than nose), and damage from an attempt to topple a statue with a concrete cutter.

### Associations with statue attacks

Statue characteristics that were significantly associated with any attack included being an older statue (ie, an earlier unveiling date), and not being made of bronze (eg, being made of marble) (Table 3). Risk of attack was relatively high for: royalty (50%), military personnel (33%), explorers (29%), and politicians (25%); but zero for sports players (0%).

**Table 3:** Characteristics of attacked (n = 28) and non-attacked (n = 95) statues (for any attacks over the “lifetime” of each statue).

Characteristics	Risk of attack in group of interest	Risk of attack in comparison group	P-value*
**Statue subject characteristics**			
Male (n = 107) (vs comparison group: female)	24.3%	12.5%	0.3171
European (n = 114) vs other ethnicities	23.7%	11.1%	0.4372
Statue subject—primary reason for fame**			
Royalty (king or queen) (n = 6) vs not royalty	50.0%	21.4%	0.1543
Military personnel (n = 21) vs other	33.3%	20.6%	0.1133
Explorer (n = 14) vs other	28.6%	22.0%	0.5823
Politician (n = 20) vs other	25.0%	22.3%	0.7798
Sports player (n = 11) vs other	0.0%	20.0%	0.0960
**Statue features**			
Statue mean age (years) in attacked statues (vs not attacked)	62.8 years (SD = 43.0)	42.3 years (SD = 45.5)	0.0365 (ANOVA)
Not made out of bronze (n = 39) (ie, marble, concrete, composite materials) vs bronze	38.5%	15.5%	0.0034
Statue subject involved in colonialism in NZ or direct harm to Māori (n = 22)	59.1%	14.9%	<0.0001

* Mid-P exact test (unless otherwise stated).

** There were no attacks on statues of: social activists (n = 7), religious leaders (n = 3), doctors (n = 2), or scientists (n = 2).

In the logistic regression analysis adjusting for statue age and material (bronze vs other material), the statues of subjects involved in colonialism or direct harm to Māori (based on our *a priori* hypothesis) were more likely to have been attacked: adjusted odds ratio (aOR) of 6.61 (95%CI: 2.30 to 19.9), compared to all other statues (Table 4). Similarly, such statues had a higher number of attacks at 1.3 vs 0.2 attacks per statue, with an adjusted rate ratio of 6.13 using Poisson regression (95%CI: 3.22 to 12.0; Table 4).

**Table 4:** Risk of attack by statue subject being involved in colonialism or direct harm to Māori, relative to other statue subjects.

Type of attacks (over the full statue’s lifetime)	Statues of subjects involved in colonialism or direct harm to Māori	Statues of other subjects	Crude ratio	Adjusted regression analysis, (95%CI) and p-value; (simultaneous adjustment for statue age and material)
Any attack	59.1% (13/22)	14.9% (15/101)	8.25 (3.06 to 23.6); p<0.0001 (odds ratio [OR])	aOR = 6.61 (2.30 to 19.9); p = 0.0005 (adjusted OR, logistic regression)
Number of attacks per statue	Mean rate = 1.27 (28 attacks on 22 statues)	Mean rate = 0.17 (17 attacks on 101 statues)	7.56 (4.18 to 14.1); p<0.001 (rate ratio [RR])	aRR = 6.13 (3.22 to 12.0); p<0.001 (adjusted RR, Poisson regression)

Additional qualitative analysis also indicated that of the attacked statues, half (50%, 14/28) had some evidence to suggest that the attack was directly motivated by the statue subject’s past associations with colonialism or direct harm to Māori (see S4 Table in S1 File for more details on each attacked statue). Other qualitative evidence is suggestive of three statue subjects who were outside this colonialism category, that were subject to attacks on anti-war grounds ie, Kitchener (South African War and First World War), Upham (Second World War) and Holyoake (Vietnam War), (S4 Table in S1 File).

## Discussion

### Main findings and interpretation

This study found that 23% of outdoor public statues of named individuals had been attacked at least once (a total of 45 attacks), with an increase in the number of attacks in the last three decades. Injuries ranged from paint attacks that can be relatively easy to repair, to decapitation and even complete statue destruction. We found that attacks appeared to be more likely if the statue subject had been involved in colonialism or direct harm to Māori. This pattern is consistent with aspects of the international pattern of statue attacks (see *Introduction*). Indeed, attacks have occurred internationally on some of the exact same statue figures in other countries beside New Zealand eg, Captain Cook in Australia [21], and King George V in South Africa [22].

This pattern was also supported by the qualitative results indicating that of the attacked statues, 50% had some documented evidence linking the statue subject to colonialism or direct harm to Māori eg, attacks on the statues of former Prime Minister Ballance [23], and George Grey [24] (S4 Table in S1 File). Nevertheless, other possible reasons for statue attacks may have been related to New Zealand’s participation in international conflicts eg, for attacks on former Prime Minister Holyoake on an anniversary of the Vietnam War. Also some of the other attacks might plausibly be more in the domain of “pranks” as per the decapitation of the statue of the poet Robbie Burns in Timaru.

The disproportionate domination of statue subjects by European males (relative to Māori, Pacific peoples, Asian peoples and women), may partly reflect historical and persisting power relations in New Zealand society. The patterns may also reflect occupational dominance of men in much of New Zealand history, particularly in the fields of politics, the military, and science/exploration. Other factors might include the role of international linkages with New Zealand having overlapping power structures with the United Kingdom (ie, the same kings and queens).

The ethnic composition of the New Zealand population keeps changing, with the ongoing growth in the relative proportions of Māori, Pacific and Asian peoples in the past century. This will also contribute to disproportionate representation, unless the addition of new statues addresses the changes. Different cultures may also favour other forms of public art and different memorials to the western conceptualisation of a statue eg, there is a renaissance of Māori art forms in recent decades that include art works such as “pou” (involving ancestor representations) as well as statues in private settings such as marae, and in urupā (Māori cemeteries) [25].

The New Zealand experience, as indicated by the data we found, has general similarities to some of the international experience, but also has distinctive differences. As elsewhere, changing political power balances and shifts in cultural beliefs have meant that some statues are seen differently than in the past [26], but this will have different effects in different places [27]. The New Zealand context will result in differences, for example, compared to royal statues in Ireland [28], or for statues of the same person in another country [12].

### Study strengths and limitations

A strength of this study was that it is the first full national survey of a sample of statues and statue attacks in any nation (to the best of our knowledge), and included field visits to all the identified outdoor statues of named individuals. This achievement was facilitated by New Zealand having a relatively small population and having detailed historical documentation with the digitalisation of historic New Zealand newspapers (ie, *Papers Past*). Our field work was also assisted by the low-quality of some statue repair work which allowed for the ready diagnosis of past injuries eg, the crude nose replacements for Queen Victoria and Earl Jellicoe (Fig 2 and S1 Fig in S1 File). Similarly for statues being left unrepaired eg, the missing nose on Robbie Burns in the town of Hokitika (Fig 2).

This study had a number of potential limitations. Firstly, we may have missed some statues with our search strategies (eg, as per the upper estimate of the sensitivity of our initial strategy at 94%). Nevertheless, in the subsequent two years since data collection was completed, we have identified no missed statues (though several new ones were unveiled subsequent to our previous cut-off date).

Secondly, we may have missed historical documentation of past attacks, as the police and various local government agencies may purposefully have tried to avoid media coverage of an attack and that the repairs may have been conducted discretely. Also, although we used cameras with telephoto capacity, we might have missed some signs of injuries or repairs of statues on tall plinths (eg, as per one example shown in S2 Fig in S1 File). We will have missed attacks where the effects of paint or other visible evidence were removed by an agency or the weather, and the attacks were not found in the media record. Thus we are likely to have underestimated the total number of attacks.

Our definitions were also somewhat conservative in that for physical attacks we did not include damage to: secondary figures which were part of an overall memorial (eg, the statue of a Māori warrior beneath a statue of George V in Rotorua was completely destroyed in a 1936 attack); and damage to non-body parts of the main statue (eg, to walking sticks etc). We also conservatively counted reports of “multiple” past attacks (eg, theft of a nose) as only “two” attacks.

Thirdly, our statistical analysis was also constrained by the relatively small number of statues (n = 123) and our limited ability to consider potential confounding variables (limited to statue age and construction material). For example, statue location might plausibly play a role in that statues located on busy streets might be more obvious than those in parks, and hence be more likely to be noticed by potential attackers. Alternately, statues on busy streets (and typically with street lighting at night) might be more difficult to attack without attracting attention by others and the police. Similarly, while plinth height might be a deterrent for some attackers, it may not deter those who can: throw “paint bombs”, lasso the head of the statue with a rope, or carry a portable ladder (eg, there are compact 9 kg ladders with telescoping components). Furthermore, the small sample size limited our capacity to determine if there was differential distribution of statue location by statue subject.

All these limitations highlight the need for further research on statues in New Zealand and internationally. We are now undertaking a survey of all military statues in outdoor public places in New Zealand, both generic and named individuals. This type of national survey could be conducted in other countries and cross-country comparisons performed. Such surveys could also inform public discussions about the appropriate role of statues in modern society and the processes for dealing with ones that are controversial.

## Conclusions

This national survey found an association between statue attacks and the role of statue subjects in colonialism or direct harm to the Indigenous population. Furthermore, the demography of the statue subjects may represent historical and current social power relationships—with under-representation of women and non-European ethnic groups.

## Supporting information

S1 File Additional methods and results for study on statue attacks. (PDF)

S2 File Data for NZ statue study in excel. (XLSX)
