# Supplementary material for: The epidemiology of attacks on statues: New Zealand as a case study
Source: PLoS One. 2021 Jun 3;16(6):e0252567. doi: 10.1371/journal.pone.0252567 (PMC8174703; doi:10.1371/journal.pone.0252567)
Supplement: S1 File — (PDF) [file pone.0252567.s001.pdf]

Supporting Information: Additional methods and results for study on statue attacks

Search strategy for statues and information on them: No detailed list of existing statues in New Zealand could be identified, so we used the following sources to help identify them: books on New Zealand sculpture [1] [2], and memorials [3-6]; a list of memorials and monuments in a New Zealand Encyclopaedia [7]; a city-specific sculpture websites [8] [9], and an international statue website [10].

To identify additional statues we conducted searches in Google Images using the following search terms (all conducted in September 2018):

- "Statue and Zealand and [city name]" for all New Zealand cities (ie, urban centres with a population of 50,000+ [~100 images each]): Auckland, Wellington, Christchurch, Hamilton, Tauranga, Napier-Hastings, Dunedin, Palmerston North, Nelson, Rotorua, Whangarei, New Plymouth, and Invercargill.
- "Statue and Zealand and [name]" for a list of 100 famous New Zealanders ranked out of 430 notable people by a panel on a television series on famous New Zealanders [11].
- "Statue and Zealand" (for all 100+ images returned)
- "Bust and Zealand" (for all 100+ images returned)
- "Bas relief" and Zealand (for all images returned)
- "Zealand statue unveiled " (for all images returned)

After the above, further searches were made using "Papers Past" [12], which has a national repository for all New Zealand newspaper issues published from 1/1/1839 to 31/12/1949. Search terms included the exact phrase of "statue unveiled" and "statue unveiling" (and similarly for "bust"). But also to identify less well documented statues of Māori, we also searched using "Maori and statue". Google Image searches and the Papers Past searches were terminated when yielding under one new statue for 30 minutes of search time with each respective search engine. For additional context on the statues, we searched Google Scholar for scholarly literature relating to statues in New Zealand (eg, [3, 13-17]).

Evaluating search strategy sensitivity: We evaluated our search strategy by conducting additional searching for all the statues in New Zealand's largest city: Auckland. This involved searches by a different co-author (the last named author) from the original searches detailed above (by the first author). The searches involved some of the same, but also additional elements to the above searches:

- Google Images, using all of: 'Auckland' 'Zealand' 'statue OR bust'
- The largest newspaper based in Auckland (the NZ Herald), using: 'statue' 'Zealand' (first 15 pages); and 'statue' 'Auckland' (all 20 pages)
- The Factiva Database for "NZ publications" and "all dates" with the terms:
    - 'statue and Auckland and unveiling' (all 69 returns)
    - 'statue and Zealand and unveiling' (all 100 returns)
- "Papers Past", using: 'Auckland' and 'statue'

This extra strategy identified no new statues (out of the 16 statues in Auckland), giving us some confidence that the original strategy was fairly sensitive. Furthermore, Papers Past appeared to typically have multiple newspaper items that documented the same statue unveiling – suggesting that this was a relatively sensitive mechanism for statue identification. Nevertheless, subsequent to the original searches, we did incidentally locate six additional statues as a result of both further internet searches (n=3), and field work (n=3) up to the last inclusion date (13 April 2019 when the statue of Lithgow was unveiled). This suggested our initial search strategy was no more than 94% sensitive (116/123).

Statue attack data: Identifying a statue as per the above search strategies frequently revealed historical information about attacks on the statue. But we also performed additional searches in Papers Past and with search terms including the words: statue and vandalism; statue and paint and damage; statue decapitation. Similarly for Google searches: Zealand and statue and decapitation/graffiti/stolen/broken.

Field data: Site visits to all the statues that were still outdoors were conducted between September 2018 and August 2019. Photographs were taken of the setting and from all sides of the statues (including of any associated plaques or information boards). In particular, evidence for past and current injuries (including attacks with paint) were closely looked for eg, in nostrils and ear canals – where removal of paint is more difficult.

Data coding and analysis: Data from internet searches and field visits were collated in an Excel file and analysed. The denominator used was for all statues unveiled at any time up to the last inclusion date (13 April 2019). This therefore included all those subsequently destroyed (n=3), stolen (n=1), or moved from outdoors and into interior settings (n=14). When a stolen statue was replaced (n=1) this was included as a new statue in the denominator.

We defined statue attacks as being where there was documentation of a historical attack (eg, in *Papers Past* or in online media reports) or from our field observations of obvious repairs or selected missing body parts (eg, noses), paint remnants, or graffiti. Not included were minor embellishments such as the addition of lipstick to the statues' lips, application of chewing gum, clothing added to the statue or having traffic cones placed on their heads. We also excluded damage that was unintentional eg, the multiple statues damaged by the 2011 Christchurch earthquake and statues destroyed by fungal infection (eg, George Grey in Greytown).

Table S1: Reasons for excluding certain types of statues with examples

Reason for exclusion	Selected example/s
Subject of the statue did not meet the following three criteria:<br>1. an **actual person**<br>2. a **non-foundational religious figure**<br>3. **part of the modern era** (from Tasman's first visit in 1642 AD)	• Generic figures: eg, many generic soldier figures on war memorials around NZ (eg, "Trooper Mackenzie" (Fairlie)); "Art Deco Woman" (Napier); "Barry" (Katikati); Rangatira (Waihi); the statue at the grave of Harry Holland (Wellington) which is a figurative one of a young man.<br>• Foundational religious figures: Virgin Mary, Jesus.<br>• Pre-European ancestors of Māori: the 18 pou with representations of Māori ancestors: "Ngā Pou O Heretaunga" (Hastings); Kupe (group statue in Wellington), Kupe (Whāingaroa).<br>• Fictional characters from books eg, Peter Pan (Dunedin).
Statue was only ever on **temporary display** outdoors	Various works by the artist Sam Mahon (eg, of Catherine Sintenie, Nick Smith); Dorothy Theomin (bust, Dunedin).
Statue is **not in a public setting** (eg, in a marae, cemetery or urupā [Māori cemetery], or school). We made this criteria to ensure that the statues were likely to represent a relatively public figure – and not be for a person who was significant just to their family, family group, or iwi (tribe).	• Statues on marae/urupā (eg, of: Maui Pomare, Tangatahara, King Mahuta, Pouawha Meihana, Henare Pohio).<br>• Statues in school grounds: Captain James Cook, James (Jack) Lovelock, St John Baptist De La Salle.<br>• Statue in a fenced stadium (ie, not routinely accessible to the public): Arthur Lydiard.<br>• Statues on private land: Richard Nixon (Nelson), Tommy Solomon (Chatham Islands), Mark Twain (Matakana), Edwin Mitchelson (Auckland), Edmund Hillary (Mt Cook – within hotel premises).<br>• Statues in cemeteries: Harry Holland (Wellington).
The main statue is to something else with the **person being secondary**	The jockey (Jim Pike) on the statue of the racehorse Phar Lap (which is the primary statue).
Statue has always been located **indoors**	Kate Sheppard (Wellington); Seddon (bust in Wellington); Tupai (bust in Wellington); Kiri Te Kanawa (Auckland); Te Heuheu Tukino IV (Horonuku) (National Park, Mt Ruapehu).
Statue is clearly **miniaturised** (ie, half-sized or less in height)	Dick Henderson with wounded solider on a donkey (Wellington).
The statue has no **three dimensionality** (in contrast to a bas relief)	Caesar Roose (Hamilton)
**Bas relief** is not full length; or is only a component of a larger statue	Jean Batten (Rotorua), Austen Deans (Mt Peel)
The work is limited to being a carved **head on a building**	Various members of Royalty (eg, High Court Building, Auckland)

Table S2: Full list of the 123 outdoor statues in public places identified in New Zealand (ordered by surname)

Surname, first name	Location and notes
Alley, Rewi	Auckland
Atkinson, Henry	Auckland
Auckland, Lord (George Eden)	Auckland
Ballance, John	Wellington
Ballance, John	Whanganui (outside Council Buildings)
Ballance, John	Whanganui (statue attacked and destroyed, Moutoa Gardens)
Batten, Jean	Auckland
Beavon, Professor Sir Donald Ward	Christchurch
Bourne, Possum	Auckland (Pukekohe)
Burns, Robert (Robbie)	Auckland
Burns, Robert (Robbie)	Dunedin
Burns, Robert (Robbie)	Hokitika (see Fig 2, main text)
Burns, Robert (Robbie)	Timaru
Byrd, Rear Admiral Richard Evelyn	Dunedin
Byrd, Rear Admiral Richard Evelyn	Wellington
Cain, Captain Henry	Timaru
Campbell, John Logan	Auckland
Carrington, Frederic	New Plymouth
Carter, Charles Rooking	Carterton
Colvin, James	Westport
Cook, Captain James	Auckland (this was originally outside a brewery but has since been moved indoors)
Cook, Captain James	Christchurch
Cook, Captain James	Gisborne (was on Kaiti Hill but was moved to a museum in 2019)
Cook, Captain James	Gisborne (river mouth location)
Cook, Captain James	Marton
Cruickshank, Dr Margaret	Waimate
Crumen, Barrett ("Russian Jack")	Masterton
Daldy, Amey	Christchurch (temporarily in storage)
Dickson, Frank	Christchurch (temporarily in storage)
Elworthy, Rt Hon Lord (Samuel Charles)	Timaru
Fantham, A.A.	Hawera (Taranaki)
Fitzgerald, James Edward	Christchurch
Fitzsimmons, Bob	Timaru
Fraser, Peter	Wellington
Freyberg, Lord Bernard	Auckland

Surname, first name	Location and notes
Gallaher, Dave	Auckland
Gandhi, Mahatma	Wellington
George V, King	Matakana (town near Warkworth)
George V, King	Rotorua
Godley, John Robert	Christchurch
Grant, John (VC)	Hawera (Taranaki)
Grey, Sir George	Auckland
Grey, Sir George	Greytown (was destroyed by fungal infection)
Grigg, John	Ashburton
Hadlee, Sir Richard John	Christchurch (temporarily in storage)
Hamilton, Captain John Fane Charles	Hamilton (see Fig 2, main text)
Herd, David	Blenheim
Hillary, Sir Edmund	Orewa
Holyoake, Sir Keith	Wellington
Isaac, Lady Diana	Christchurch (temporarily in storage)
Jellicoe, Earl John	Invercargill (see Fig S1)
Joll, Thomas Langdon	Okaiawa (Taranaki)
Jones, Sir Michael	Auckland (see Fig S3)
Jordan, Canon Charles	Tauranga
Kipling, Rudyard	Christchurch
Kitchener, Field Marshal Horatio Herbert	Auckland (destroyed in an attack)
Kitchener, Field Marshal Horatio Herbert	Invercargill
Laurent, Henry (VC)	Hawera (Taranaki)
Lindauer, Gottfried	Woodville
Lithgow, Alex	Invercargill
Little, Dr Charles	Waikari (North Canterbury) (see Fig S4)
Locke, Elsie	Christchurch (temporarily in storage)
Luney, Charles	Christchurch (temporarily in storage)
Macandrew, James	Dunedin
Mackenzie, James	Fairlie (South Canterbury)
Mahy, Margaret	Christchurch (temporarily in storage)
Malfroy, Camille	Rotorua
Malone, Lieutenant Colonel William	Stratford (Taranaki)
Mangakāhia, Meri Te Tai	Christchurch (part of suffragette bas-relief)
Mansfield, Katherine	Wellington
Masters, Joseph	Masterton
McKinnon, Quintin	Te Anau
Meads, Sir Colin	Te Kuiti
Meryon, Charles	Akaroa

Surname, first name	Location and notes
Monro, Charles	Palmerston North
Moore-Jones, Horace	Hamilton
Moorhouse, William Sefton	Christchurch
Morison, Harriet	Christchurch (part of suffragette bas-relief)
Munro, Burt	Invercargill
Nelson, William	Hastings
Nicholas, Henry James (VC)	Christchurch
Nicol, Helen	Christchurch (part of suffragette bas-relief)
O’Regan, Sir Tipene	Christchurch (temporarily in storage)
Plimmer, John	Wellington
Rees, William Gilbert	Queenstown
Robinson, Sir Dove-Myer	Auckland
Rolleston, William	Christchurch
Ruskin, John	Christchurch
Russell, Sir Andrew	Hastings
Rutherford, Ernest	Brightwater (Nelson)
Scott, Sir Robert Falcon	Christchurch
Seddon, Richard (Dick)	Hokitika
Seddon, Richard (Dick)	Wellington
Sheppard, Kate	Christchurch (part of suffragette bas-relief)
Snell, Sir Peter	Opunake (Taranaki)
Snell, Sir Peter	Whanganui
Stewart, Sir Robertson	Christchurch (temporarily in storage)
Stuart, Rev Dr Donald	Dunedin
Sutton, Bill	Christchurch (temporarily in storage)
Tait, Admiral Sir Gordon	Timaru (stolen)
Tait, Admiral Sir Gordon	Timaru (replacement of stolen one)
Tait, Sir Angus	Christchurch (temporarily in storage)
Tasman, Abel	Nelson (see Fig 2, main text)
Te Awe Awe, Te Peeti	Palmerston North
Te Rangihiwinui, Te Keepa	Whanganui (see Fig S2)
Te Rauparaha	Otaki
Te Wainohu, Henare Wepiha	Wairoa
Thomson, John Turnbull	Ranfurly (Otago)
Upham, Charles (VC)	Amberley (North Canterbury)
Victoria, Queen	Auckland
Victoria, Queen	Christchurch
Victoria, Queen	Dunedin (see Fig 2, main text)
Victoria, Queen	Wellington

Surname, first name	Location and notes
Wakefield, Edward Gibbon	Wellington
Walker, Sir John	Auckland
Ward, Sir Joseph	Bluff (Southland)
Warren, Sir Miles	Christchurch (temporarily in storage)
Wellington, Duke of	Wellington
Wells, Ada	Christchurch (part of suffragette bas-relief)
Whakarua, Herewini	Whanganui (see Fig S2)
Worsley, Frank	Akaroa
Wylie, Fred W.	Rotorua
Young, Nicholas ("Young Nick")	Gisborne

Table S3. *A priori* identification of statue subjects in terms of colonialism or direct harm to Māori

Name of the statue subject	Reason for fame	Details
Ballance, John	Prime Minister of NZ	He was involved in the NZ Wars [15], which were land wars between colonial forces and Māori. While he "saw some limited action with the Wanganui Cavalry Volunteers" [18], his military rank is assumed to have been low. Although he became Minister of Defence and Prime Minister, these were after the time of the NZ Wars.
Cook, James [Captain]	Explorer	He was the expedition leader when some local Māori were killed by his crew at the time of his visit to Gisborne in 1769. This European contact also symbolises the start of the loss of Māori land to Europeans in the subsequent century and longer. Within the Royal Navy he held the rank of lieutenant during his first voyage to the Pacific. He was then promoted one rank to commander for his second voyage to the Pacific. He was then promoted one rank to "Post-captain" for his third voyage.
George V [King]	King of the UK and the British Dominions (including NZ)	He was King from 1910 to 1936, a time when the British Crown substantially ignored its Treaty of Waitangi obligations. His deficient role is given emphasis as he (as the Crown) was the direct Treaty partner with Māori in NZ. Indeed, during his reign in 1914 and 1924 deputations of Māori travelled to England to take petitions based on the Treaty to the British monarch and the British Government [19]. He had a powerful figurehead role during WW1 and he was very active in visiting the troops. In terms of military ranks for NZ, the highest ranks are taken by royalty eg, the admiral of the fleet (navy) and field marshal (army).
Grey, George [Sir]	Governor of NZ; Premier of NZ	He was involved in the NZ Wars where Māori land was taken [13]. His position as Governor of New Zealand, although a mainly political one, can be regarded as equivalent to a high ranking military one during the time of the NZ Wars.
Hamilton, John [Captain]	Military leader/ City of Hamilton named after him	He was involved in the Battle of Gate Pā (NZ Wars) where Māori were killed. His highest rank was as a Commander in the navy.
Tasman, Abel	Explorer and navigator	He was the leader when Māori were killed in an encounter with his ships. His highest rank was a Commander.
Te Awe Awe, Te Peeti	Māori Chief involved in the NZ Wars and also land disputes	He was part of an alliance with the Crown in the Taranaki Campaign in the NZ Wars [20]. His military rank status was not identified in our literature search, but we consider it likely to be at least officer level equivalency given his status as a chief. He was also involved in land disputes with other Māori tribes [20].

Name of the statue subject	Reason for fame	Details
Te Rangihiwinui, Te Keepa	Māori Chief involved in the NZ Wars	He was a military leader in alliances with the Crown in the NZ Wars [21]. His highest rank was Major. See Fig S2.
Te Rauparaha	Māori Chief/military leader	He was involved in the “Musket Wars” with conflict against other Māori tribes. He can be considered to have been of a high military rank given his military leadership of his tribe, Ngati Toa [22].
Victoria, Queen	Queen of the UK and the British Dominions (including NZ)	She was Queen and was symbolic of the British colonial empire. In particular, she was Queen during the NZ Wars and during a period when the Treaty of Waitangi was dishonoured by the Crown [23]. Her deficient role is given emphasis as she (as the Crown) was the direct Treaty partner with Māori in NZ. Indeed, during her reign in 1882 and 1884 deputations of Māori travelled to England to take petitions based on the Treaty to the British monarch and the British Government [19]. See under George V as per the military status of Royalty.
Wakefield, Edward Gibbon	A founder of the European colonisation of NZ	He was a strong promoter of colonisation of NZ and in general, with an actual “colonisation theory” [24]. Another aspect of his history was that he was imprisoned (in England) for abducting a 15-year-old schoolgirl [24].
Young, Nick (Nicholas)	First person on Cook’s ship to sight NZ	He was part of Captain Cook’s crew (part of the Royal Navy) when some local Māori were killed by this crew at the time of Cook’s visit to Gisborne. This European contact also symbolises the start of the loss of Māori land to Europeans in the subsequent century and longer.

Table S4: Specific details on the 28 statues with intentional attacks (physical damage, paint or theft)

Name of the memorialised person (locality)	Reason for fame	Classified as involved in colonisation/harm to Māori	Nature of injuries/ damage (dates)	Possible reason/s for damage	Outcomes
Atkinson, Henry (Auckland)	Engineer/ philanthropist	No	Repeated thefts of his nose (unknown dates)	Unknown – possibly pranks	Repaired and moved from a hilltop location to a more urban location
Ballance, John (Wellington)	Prime Minister of NZ	Yes	Decapitation and the words “Pākaitore is Māori land” spray painted on it (in 1995) [15]	Probably related to loss of Māori land and his involvement in the NZ Wars against Māori [15] (see Table S3)	Head replaced and paint removed
Ballance, John (Moutoa Gardens, Whanganui)	Prime Minister of NZ	Yes	Decapitation in both 1994 and 1995 [15]. Then after a further period the torso disappeared (1995)	See above	Statue never replaced at this site but a bronze one was subsequently unveiled elsewhere in the city (see below)

Name of the memorialised person (locality)	Reason for fame	Classified as involved in colonisation/harm to Māori	Nature of injuries/ damage (dates)	Possible reason/s for damage	Outcomes
Ballance, John (Whanganui – Council Buildings)	Prime Minister of NZ	Yes	Foot stolen	See above	Foot replaced. "When the statue was sent back to the sculptor for repairs, it was discovered it had not been cast properly and needed to be completely rebuilt" [25].
Burns, Robert (Timaru)	Scottish poet	No	Decapitation (2012)	Unknown – possibly a prank	Head replaced
Burns, Robert (Hokitika)	Scottish poet	No	Missing nose and signs of previous repair (Fig 2, main text); Repairs to hand and foot evident.	Unknown – possibly a prank	Nose still missing with wires visible in nasal area; poor quality repair to his hand.
Cook, James [Captain] (Kaiti Hill, Gisborne)	Explorer (with his expedition first landing in NZ in the Gisborne area)	Yes	Multiple paint attacks (eg, red paint on face, white bikini in past decade)	Probably associated with local Māori killed at the time of Cook's visit; possibly also symbolising the start of the loss of Māori land to Europeans (see Table S3)	Cleaned – but in 2019 the relevant City Council moved the statue to a local museum
Cook, James [Captain] (foreshore, Gisborne)	Explorer (with his expedition first landing in NZ in the Gisborne area)	Yes	Multiple attacks of red paint to face and mid-section (last decade, eg, 2016)	See above	Paint removed
Cook, James (Christchurch)	Explorer	Yes	Graffiti (field observation) – possibly tagging	See above	Unknown
Crumen, Barrett ("Russian Jack") (Masterton)	Local character/ itinerant labourer (swagger)	No	Likely paint attack given remains of paint on the face (bronze statue) found in field observations	Unknown	Paint appears to have been largely removed
George V [King] (Matakana)	King of the UK and the British Dominions (including NZ)	Yes	Decapitated a total of 5 times	Possibly due to the attitude of the Crown to Māori (see Table S3).	Head replaced each time (combined with other restoration work)

Name of the memorialised person (locality)	Reason for fame	Classified as involved in colonisation/harm to Māori	Nature of injuries/ damage (dates)	Possible reason/s for damage	Outcomes
				Possibly related to opposition to World War One, or possibly just pranks.	
Grey, George [Sir] (Auckland)	Governor of NZ; Premier of NZ	Yes	Paint attack (1952), subsequent theft of hands in subsequent decade or so; Decapitation (1987)	Involvement in the NZ Wars where Māori land was taken [13] (see Table S3)	Repairs conducted; Head never recovered and so a new one produced
Hamilton, John [Captain] (Hamilton)	Military leader/ City of Hamilton named after him	Yes	Paint attack plus blows from a hammer which appear to have caused indentations on the nose (2018) (Fig 2 main text). Other signs of graffiti on face and back	Involved in the Battle of Gate Pā (NZ Wars) where Māori were killed (as reported by the perpetrator in the media) (see also Table S3)	Paint largely removed. Indentations and graffiti (black marker pen) visible in December 2018 (field visit)
Holyoake, Keith (Wellington)	Prime Minister	No	Paint attack (red paint on both hands) in 1998 [26]	Reported to be "an apparent protest against Vietnam War commemorations occurring at the time" (he was involved in sending NZ troops to Vietnam [27])	Paint removed
Jellicoe, John Rushworth [Earl] (Invercargill)	Military leader and Governor General of NZ	No	Missing a nose (see Fig S1), probably due to vandalism (2008)	Unknown – possibly a prank; possibly an anti-war statement as he was a military leader in the First World War	After having no nose for 10 years, it was replaced in 2018 – albeit with suboptimal alignment (field observations)
Kipling, Rudyard (Christchurch)	Writer	No	Blue paint marks on field observations	Unknown – possibly a prank	Unknown
Kitchener, Horatio Herbert [Field Marshal] (Auckland)	Military leader in World War One (WW1)	No	Decapitation – probably with an axe (1931)	Unknown – but possibly related to First World War grievances or his establishment of concentration camps in the South African War	The rest of the statue was taken down and never replaced

Name of the memorialised person (locality)	Reason for fame	Classified as involved in colonisation/harm to Māori	Nature of injuries/ damage (dates)	Possible reason/s for damage	Outcomes
Kitchener, Horatio Herbert [Field Marshal] (Invercargill)	Military leader in World War One (WW1)	No	Nugget (shoe polish) smeared over face and other parts (1932)	See above	Cleaned
Plimmer, John (Wellington)	Business leader	No	Paint attack – looking like tagging marks (2011) and possibly related to an All Blacks Parade in the city a few hours later	Unknown – possibly a prank	Paint removed
Ruskin, John (Christchurch)	Art critic, writer, artist, social activist	No	Missing nose at time of field observations	Unknown – possibly a prank	Unknown
Tait, Gordon [Admiral Sir] (Timaru)	Military leader	No	Stolen and never recovered	Unknown – possibly for the value of the scrap metal	A new bust was made and more securely fastened than previously
Te Awe Awe, Te Peeti (Palmerston North)	Māori Chief involved in the NZ Wars and also land sales (while also opposing the colonial government)	Yes	Red paint attack (2018) with message painted on the surrounding ground: “Kawana land stolen”	Probably inter-tribal issues around Māori land loss (ie, "Kawana land stolen") (see also Table S3)	Paint removed
Upham, Charles	Military hero (two Victoria Crosses)	No	Attempt at cutting it down at the base with a concrete cutter (1998)	Anti-military action by a perpetrator who had a long history of anti-military protests in NZ [28]	Fully repaired. Perpetrator fined $600
Victoria, [Queen] (Auckland)	Queen of the UK and the British Dominions (including NZ)	Yes	Paint attack in 1952 in conjunction with one on the nearby statue of Grey	Probably a political component with the words “Ravager of the Māoris” written on the nearby statue of Grey in the simultaneous attack [15] (see also Table S3)	Repaired
Victoria, [Queen] (Dunedin)	Queen of the UK and the British	Yes	Nose smashed and black paint attack (mid-1990s); damage	Unknown – some might be pranks to place traffic cones on her head (but	Repairs to nose (Fig 2, main text) and fingers although there is residual

**Name of the memorialised person (locality)**	**Reason for fame**	**Classified as involved in colonisation/harm to Māori**	**Nature of injuries/ damage (dates)**	**Possible reason/s for damage**	**Outcomes**
	Dominions (including NZ)		to fingers (unknown date)	see also the row above and Table S3)	poor alignment of the nose repair; Paint removed
Wakefield, Edward Gibbon (Wellington)	A founder of the European colonisation of NZ	Yes	Indentations to nose, cheeks, brow, and an eyebrow (unknown date)	Possibly relating to his role of a promoter of colonisation (see Table S3), (with this more likely than his imprisonment for child abduction)	Remains unrepaired
Wellington, [Duke of] (Wellington)	Military leader (Battle of Waterloo in 1815) and a NZ city named after him	No	Indentations to nose, cheek, brow, eyebrow and chin (unknown date). Signs of gold paint on the face and in ear (unknown date)	Unknown – but he could possibly have been identified with colonialism given the NZ capital city is named after him. His statue is co-located with that of Wakefield (see directly above)	Remains unrepaired except for likely removal of paint
Young, Nicholas	First of Captain James Cook's crew to see New Zealand	Yes	Paint attack in 2016	Probably related to the attacks on the two statues of Captain Cook around this time which are also in Gisborne (see above and Table S3)	Paint removed

Fig S1: Marble statue of the military leader Earl Jellicoe (Invercargill City) showing a replacement nose that was preceded by 10 years of having no nose (photo: first author 2019)

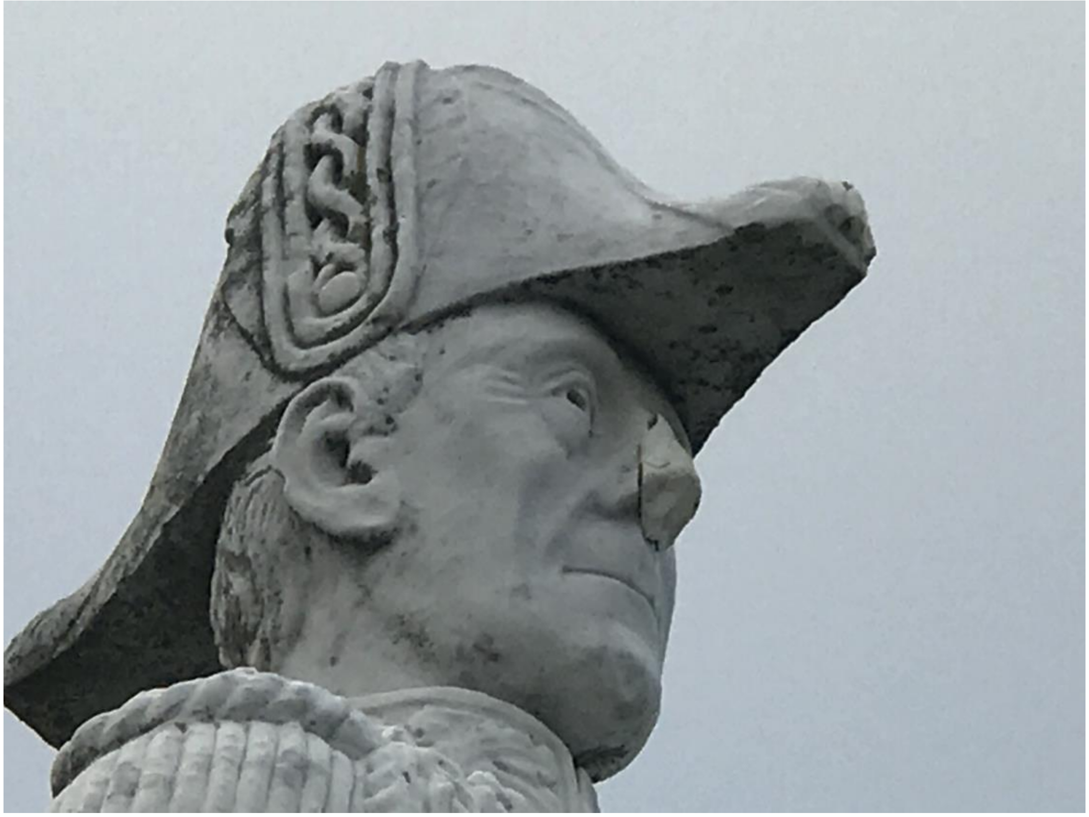

Fig S2: Marble statue of the Māori soldier Herewini Whakarua (Whanganui) on a high plinth which made close examination of the statue more difficult in this study. The statue of Te Keepa Te Rangihiwinui is in the background (photo: second author 2020)

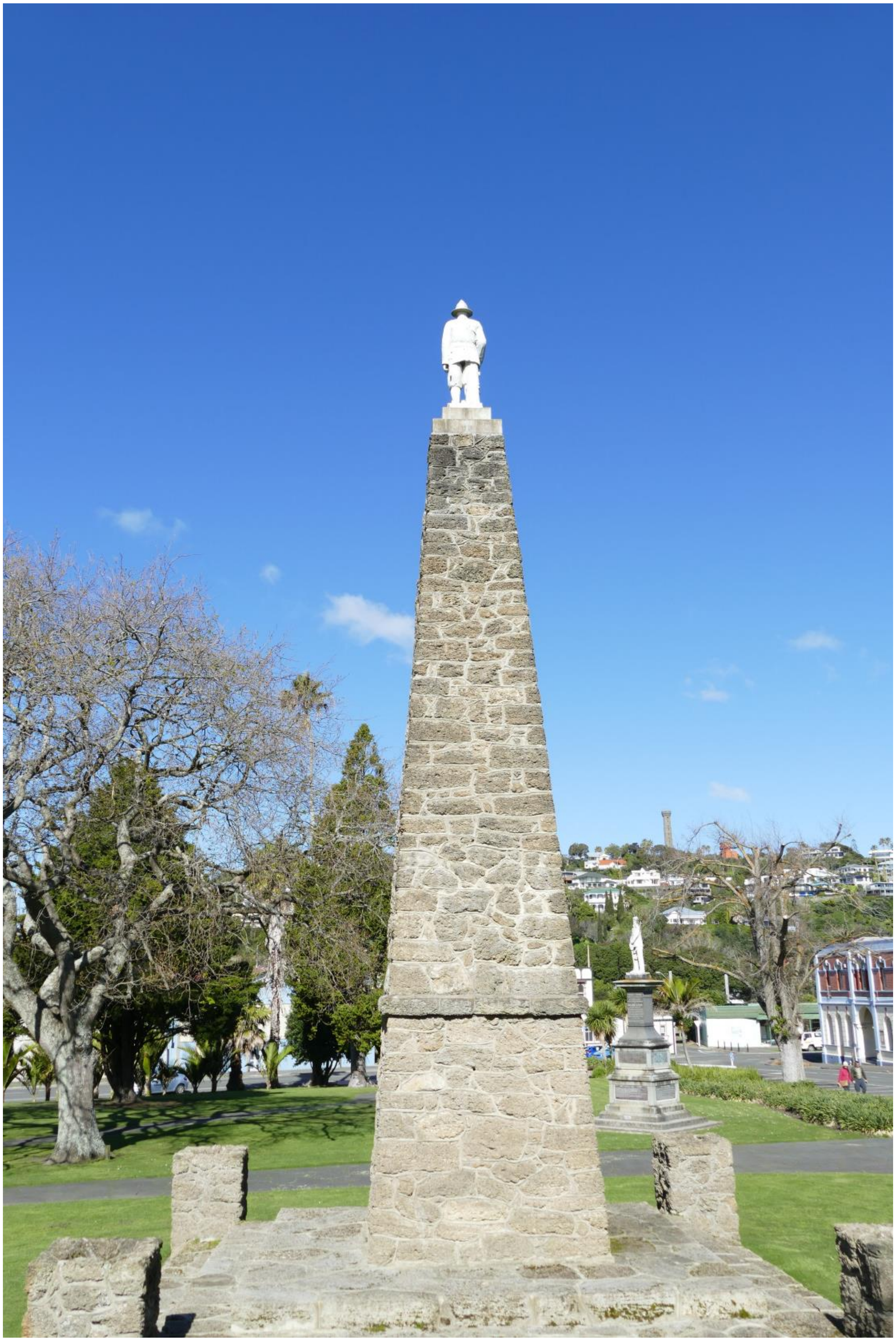

Fig S3: Statue of the rugby hero Sir Michael Jones (Auckland), the only statue of a person with Pacific peoples ethnicity identified in the survey (photo: first author 2019).

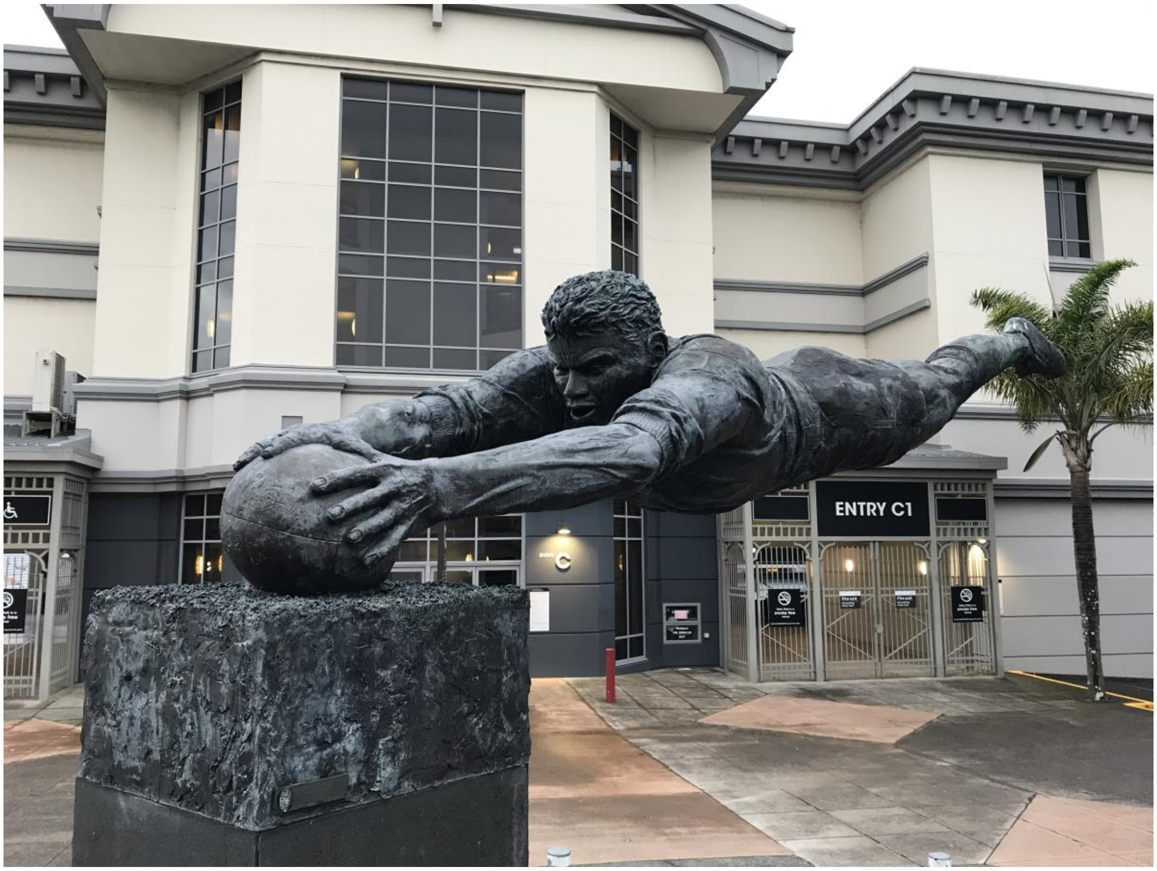

Fig S4: Statue of a local doctor (Dr Charles Little, Waikari) with evidence of lichen growth (photo: second author 2019)

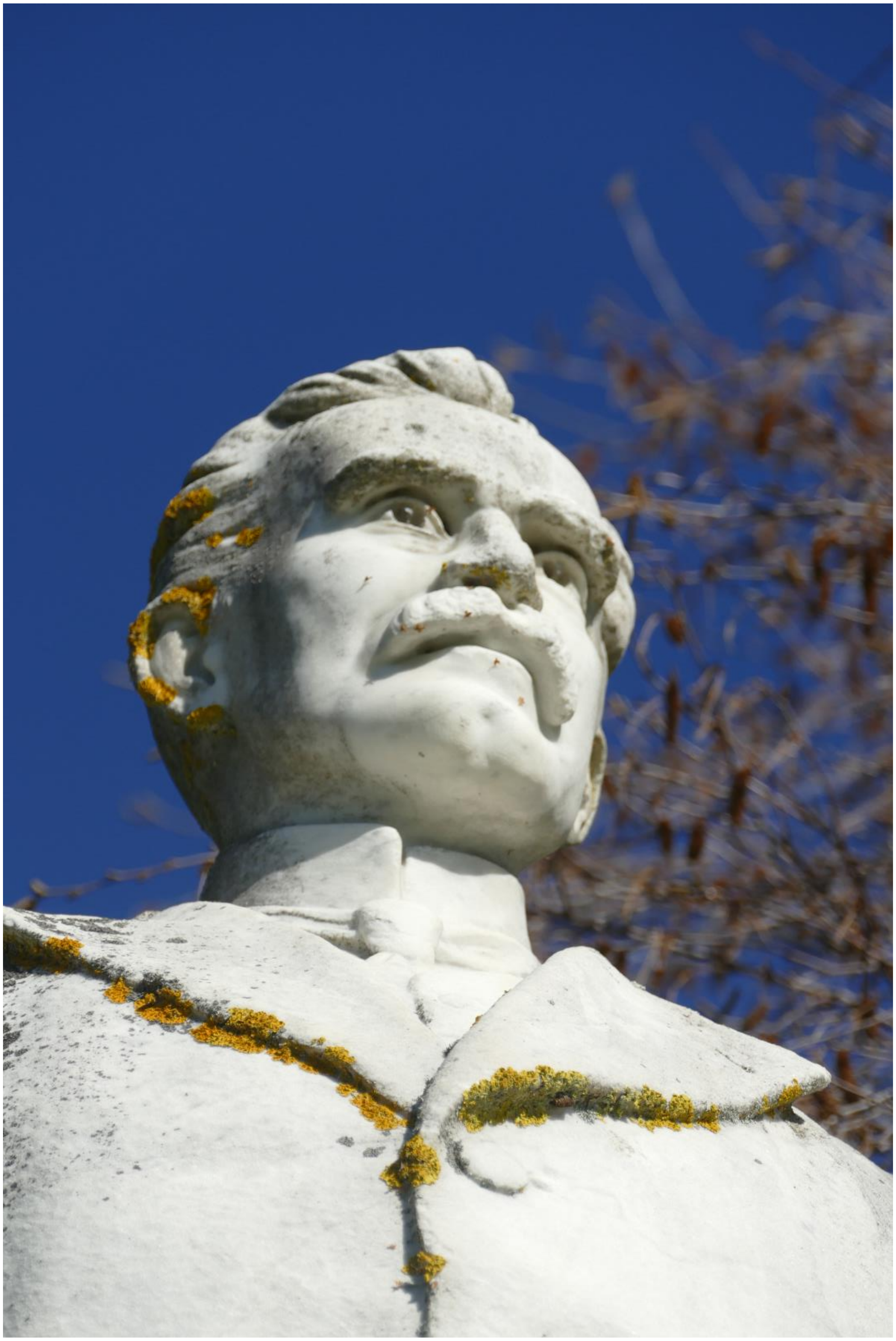

References

1. Dunn M. New Zealand sculpture: A history. Auckland: Auckland University Press; 2002.
2. Harper J, Lister A, Connew B. Wellington, A City for Sculpture. Wellington: Victoria University Press; 2007.
3. Maclean C, Phillips J. The sorrow and the pride: New Zealand war memorials. Wellington, Historical Branch with GP Books, 1990.
4. Phillips J. To the Memory: New Zealand’s War Memorials. Nelson: Potton & Burton; 2016.
5. Aberhart L. ANZAC. Wellington: Victoria University Press; 2014.
6. Sumartojo S, Wellings B. Nation, Memory, and Great War Commemoration: Mobilizing the Past in Europe, Australia and New Zealand. 2014. Bern: Peter Lang AG, International Academic Publishers, 2014
7. Phillips J. 'Memorials and monuments', Te Ara - the Encyclopedia of New Zealand. (Published 20 June 2012, updated 26 March 2015). http://www.TeAra.govt.nz/en/memorials-and-monuments (accessed 20 September 2018).
8. Wellington City Council. Sculptures & Memorials. https://wellington.govt.nz/services/community-and-culture/arts/outdoor-public-art/sculptures-and-memorials (accessed 20 September).
9. Christchurch City Libraries. Christchurch statues. https://my.christchurchcitylibraries.com/statues/ (accessed 21 September 2018).
10. van der Krogt R, Peter van der Krogt P. Statues - Hither & Thither: New Zealand (last update 24 September 2018. http://vanderkrogt.net/statues/land.php?land=NZ&webpage=ST&page=1.
11. Wikipedia. New Zealand's Top 100 History Makers. https://en.m.wikipedia.org/wiki/New_Zealand%27s_Top_100_History_Makers (accessed 21 September 2018).
12. National Library of New Zealand. Papers Past: Newspapers. https://paperspast.natlib.govt.nz/newspapers (accessed 30 September 2018).
13. Stocker M. “Director of the Canoe”: The Auckland Statue of Sir George Grey. Melbourne Art Journal. 2009;11-12:57-8.
14. Buchanan R. Why Gandhi doesn't belong at Wellington Railway Station. Journal of Social History. 2011;44(4):1077-93.
15. Morris E. Men alone, in bronze and stone: A tale of two statues. Journal of New Zealand Studies. 2012;NS13:62-76.
16. Stocker M. ‘A token of their love’: Queen Victoria Memorials in New Zealand. 19: Interdisciplinary Studies in the Long Nineteenth Century, 2016(22). DOI: http://doi.org/10.16995/ntn.724.
17. Wilson N, Ferguson C, Rice G, Baker MG, Schrader B, Clement C, et al. Remembering the 1918 influenza pandemic: national survey of memorials and scope for enhancing educational value around pandemic preparedness. N Z Med J. 2017;130(1465):53-70. Epub 2017/11/10. PubMed PMID: 29121624.
18. McIvor T. 'Ballance, John', Dictionary of New Zealand Biography, first published in 1993. Te Ara - the Encyclopedia of New Zealand, https://teara.govt.nz/en/biographies/2b5/ballance-john (accessed 22 October 2019).

19. Orange C. 'Treaty of Waitangi - Māori responses to the treaty – 1880 to 1900', Te Ara - the Encyclopedia of New Zealand, http://www.TeAra.govt.nz/en/treaty-of-waitangi/page-5 (accessed 22 October 2019).
20. Durie M. 'Te Aweawe, Te Peeti', Dictionary of New Zealand Biography, first published in 1990. Te Ara - the Encyclopedia of New Zealand, https://teara.govt.nz/en/biographies/1t27/te-aweawe-te-peeti (accessed 22 October 2019).
21. Dreaver A. 'Te Rangihiwinui, Te Keepa', Dictionary of New Zealand Biography, first published in 1990. Te Ara - the Encyclopedia of New Zealand, https://teara.govt.nz/en/biographies/1t64/te-rangihiwinui-te-keepa (accessed 22 October 2019).
22. Oliver S. 'Te Rauparaha', Dictionary of New Zealand Biography, first published in 1990. Te Ara - the Encyclopedia of New Zealand, https://teara.govt.nz/en/biographies/1t74/te-rauparaha (accessed 22 October 2019).
23. Orange C. 'Treaty of Waitangi - Dishonouring the treaty – 1860 to 1880', Te Ara - the Encyclopedia of New Zealand, http://www.TeAra.govt.nz/en/treaty-of-waitangi/page-4 (accessed 22 October 2019).
24. Fairburn M. 'Wakefield, Edward Gibbon', Dictionary of New Zealand Biography, first published in 1990. Te Ara - the Encyclopedia of New Zealand, https://teara.govt.nz/en/biographies/1w4/wakefield-edward-gibbon (accessed 22 October 2019).
25. Emerson A-M. Ballance takes up old spot again Wanganui Chronicle 2011;(1 December) https://wwwnzheraldconz/wanganui-chronicle/news/articlecfm?c_id=1503426&objectid=11047799.
26. Head J. Statue of former Prime Minister Keith Holyoake with hands painted red, Molesworth Street, Thorndon, Wellington - Photograph taken by Jo Head, [ca 6 May 1998]. Collections of the Alexander Turnbull Library. Reference Number: EP/1998/1321/19-F. http://mp.natlib.govt.nz/detail/?id=41308&l=en.
27. McGibbon I. 'Asian conflicts - Vietnam War', Te Ara - the Encyclopedia of New Zealand, http://www.TeAra.govt.nz/en/asian-conflicts/page-5 (accessed 22 October 2019).
28. Henzell J. Religious eccentric wins right of appeal. The Press. 6 June, 2007.
